# Host-pathogen interaction during mechanical ventilation: systemic or compartmentalized response?

**DOI:** 10.1186/s13054-019-2410-0

**Published:** 2019-06-14

**Authors:** Sean Keane, Ignacio Martin-Loeches

**Affiliations:** 1 Department of Anaesthesia and Critical Care Medicine, St. James’s Hospital, Dublin 8, Ireland; 2 Multidisciplinary Intensive Care Research Organization (MICRO), St James’s Hospital, Dublin 8, Ireland; 3 Pulmonary Intensive Care Unit, Respiratory Institute, Hospital Clinic of Barcelona, IDIBAPS, University of Barcelona, Barcelona, Spain

**Keywords:** Ventilator-associated pneumonia, Ventilator-associated tracheobronchitis, Continuum, Compartmentalized, De-compartmentalized, Intensive care unit, Mechanical ventilation

## Abstract

Patients admitted to the intensive care unit (ICU) often require invasive mechanical ventilation. Ventilator-associated lower respiratory tract infections (VA-LRTI), either ventilator-associated tracheobronchitis (VAT) or ventilator-associated pneumonia (VAP), are the most common complication among this patient cohort. VAT and VAP are currently diagnosed and treated as separate entities, viewed as binary disease elements despite an inherent subjectivity in distinguishing them clinically. This paper describes a new approach to pulmonary infections in critically ill patients. Our conjecture is that the host-pathogen interaction during mechanical ventilation determines a local compartmentalized or systemic de-compartmentalized response, based on host immunity and inflammation, and the pathogenic potential of the infecting organism. This compartmentalized or de-compartmentalized response establishes disease severity along a continuum of colonization, VAT or VAP. This change in approach is underpinned by the dissemination hypothesis, which acknowledges the role of immune and inflammatory systems in determining host response to pathogenic organisms in the lower respiratory tract. Those with intact immune and inflammatory pathways may limit infection to a compartmentalized VAT, while immunosuppressed mechanically ventilated patients are at greater risk of a de-compartmentalized VAP. Taking this model from the realm of theory to the bedside will require a greater understanding of inflammatory and immune pathways, and the development of novel disease-specific biomarkers and diagnostic techniques. Advances will lead to early initiation of optimal bespoke antimicrobial therapy, where the intensity and duration of therapy are tailored to clinical, immune and biomarker response. This approach will benefit towards a personalized treatment.

## Background

Patients admitted to the intensive care unit (ICU) often require organ support. Invasive mechanical ventilation, while a widely acknowledged lifesaving lung support, may be associated with deleterious effects. Ventilator-associated lower respiratory tract infections (VA-LRTI), either ventilator-associated tracheobronchitis (VAT) or ventilator-associated pneumonia (VAP), are the most common complications encountered during invasive mechanical ventilation.

VAP is a well-described and widely studied clinical entity and is linked to enhanced morbidity and mortality globally [1, 2]. Significant published work exists on the prevention, diagnosis, treatment and impact of VAP in critically ill patients, and established international guidelines outline an optimal approach to care [3, 4]. A growing body of research shows that VAT exerts a similar burden on critical care resources as VAP, but with no increase in attributable death [5–8]. Therefore, VAT is an important clinical entity that has been under-appreciated among some clinicians and researchers, evidenced by the absence of a gold standard definition and clear management guidelines.

VAT and VAP are currently diagnosed and treated as separate entities, viewed as binary disease elements despite an inherent subjectivity in distinguishing them clinically. While 1 week of treatment is currently recommended for VAP, it is still being discussed if treatment for VAT is necessary, and if so for how long.

### Colonization, VAT and VAP—a continuum

A new approach conceives colonization, VAT and VAP as a continuum of lower respiratory tract inflammation and infection. Colonization is the microbiological growth of potentially pathogenic microorganisms in tracheobronchial samples, endotracheal aspirates (ETA) or bronchoalveolar lavage (BAL), with no features of systemic infection. VAT represents an intermediate process between colonization and VAP. No gold standard definition exists for VAT, and this has led to different diagnostic criteria being applied. The Center for Disease Control (CDC) defines VAT as an absence of pneumonia on the chest radiograph (CXR) and at least two of the following: fever (> 38 °C), cough, new or increased production of sputum, rhonchi and wheezing, or bronchospasm. Additionally, the culture of tracheobronchial secretions obtained by ETA or bronchoscopic technique should be positive [9]. In more recent years, an updated definition has been developed and introduced into clinical practice. Along with absent pulmonary infiltrates on chest radiography, VAT requires the presence of at least two of body temperature > 38.5 °C or < 36.5 °C, leucocyte count > 12,000 cells per μL or < 4000 cells per μL, and purulent ETA or BAL. In addition, VAT must be microbiologically confirmed by the growth of > 10^5^ or > 10^4^ colony-forming units (CFU) per mL of a potentially pathogenic microorganism in the ETA or BAL respectively [8, 10–12]. Clinical and microbiological findings are combined to diagnose VAT, and a diagnosis cannot be made when they occur independently of each other.

VAT and VAP have similar diagnostic criteria and are distinguished by the presence or absence of pulmonary infiltrates on CXR. Making a differential diagnosis on the basis of CXR is difficult [13–15]. Occasionally, CXR is negative but computed tomography (CT) suggests pneumonia. Upchurch et al. conducted a multicentre observational study in the USA in over 2000 patients with pneumonia visualized with either CXR or CT. Among the 748 patients who underwent both CXR and CT, 87% had pneumonia on both imaging studies whereas 9% and 4% had pneumonia only on CT or on CXR respectively [16]. Chest portable radiograph remains a mandatory component for VAP diagnosis in critically ill patients; similarly to the clinical criteria, it does either have some problems with both specificity and sensitivity. Position, poor-quality films, etc. can further compromise the accuracy of chest X-rays in daily clinical practice [17, 18].

### Immune response along the continuum

We propose a radically different approach to the diagnosis of pulmonary infections in critically ill patients. Our conjecture is that the host-pathogen interaction during mechanical ventilation determines a local compartmentalized or systemic de-compartmentalized response, based on host immunity and inflammation, and the pathogenic potential of the infecting organism. This compartmentalized or de-compartmentalized response establishes disease severity along a continuum of colonization, VAT or VAP.

**Fig. 1:**
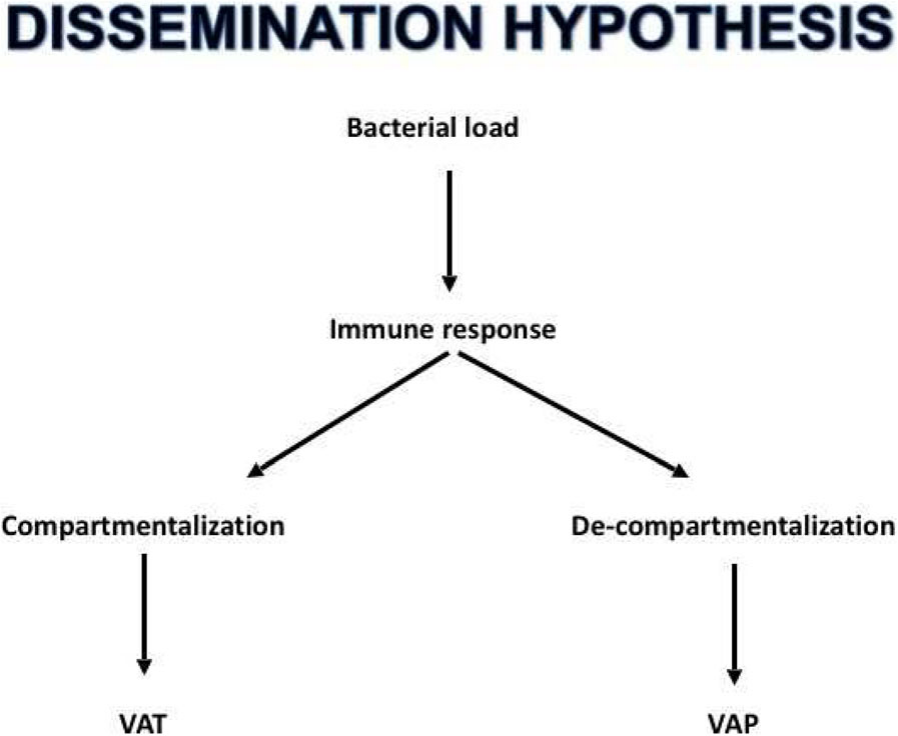
Dissemination hypothesis

This approach requires a fundamental change in how we view respiratory infections in invasive mechanically ventilated patients, from the current binary model to a continuum determination. This paradigm shift is underpinned by a dissemination hypothesis, which acknowledges the role of the immune and inflammatory systems in determining host response to pathogenic organisms in the lower respiratory tract. Those with intact immune and inflammatory pathways may limit infection to a compartmentalized VAT, while immunosuppressed mechanically ventilated patients are at greater risk of a de-compartmentalized local immune VAP (Fig. 1).

Bringing the dissemination model from the realm of theory to the bedside will require a greater understanding of inflammatory and immune pathways, and the development of novel disease-specific biomarkers and diagnostic techniques to track host response to pathogenic organisms. While a knowledge gap currently exists in this area, work is on-going that will identify these pathways and devise specific markers linked to both VAT and VAP, with subsequent incorporation into diagnostic, therapeutic and predictive algorithms.

### Systemic biomarkers

Biomarkers are proposed to have a role in the identification of new infections, distinguishing infection from inflammation, predicting severity on an individual basis, and tracking response to therapy. Despite growing research interests, biomarkers have so far failed to meet these identified needs [19, 20]. Clinically useful biomarkers for respiratory tract infections are lacking.

The use of systemic inflammation markers, such as C-reactive protein (CRP), erythrocyte sedimentation rate (ESR) and white blood cell count (WBC), is of limited utility in current clinical practice due to their poor sensitivity and specificity. Procalcitonin (PCT) has also been introduced as a valuable biomarker for bacterial infection detection. However, all of the aforementioned “traditional inflammatory biomarkers” are still far from being both specific and sensitive in VAP [21]. Potential biomarkers to inform clinical decision-making have been assessed across a number of studies [22–26]. Again, PCT, CRP and soluble triggering receptor expressed on myeloid cells (sTREM-1) are those most commonly studied, and conclusions regarding usefulness are often conflicting. More recently, TNF-receptor 1 (TNFRI) and granulocyte colony-stimulating factor (GCSF) have been discovered as promising optimal biomarkers at the day of VAP diagnosis (ROC-AUC of 0.96, with excellent sensitivity). Moreover, TNFR1 has shown also as a targeted inhibition of proinflammatory molecules by a selective antagonist of TNFR1 (GSK1995057) using a novel domain antibody (dAb) [27].

In the absence of specific biomarkers, therapy is generally empiric with little ability to instigate personalized therapy. Patients are diagnosed according to algorithms and treated by the protocol. Antimicrobial therapy is excessive for some, but inadequate for others. Effective antimicrobial stewardship programmes are impeded by a lack of accurate information regarding the appropriateness of instituted antimicrobial therapy. This may contribute to overuse of antimicrobial, inappropriate duration of therapy and emergence of resistant organisms [21, 28].

As a new model of respiratory infection, there are few studies evaluating the role of biomarkers to distinguish colonization, VAT and VAP. Extrapolated findings suggest CRP is static between VAT and subsequent development of VAP, while PCT demonstrated a significant rise as the continuum progressed [26]. The potential for PCT to accurately define the progression to infective pulmonary infiltrates is also inferred from studies in COPD and post-lung transplant patients [29, 30].

In the future, disease-specific biomarkers may enable accurate recognition of the compartmentalized immune response in lower respiratory infections, defining progression through colonization, VAT and VAP and tracking response to antimicrobial therapy.

### Is immunoparalysis a right target for physiopathology understanding in VA-LRTI?

Critically ill patients are considered to exist in a state of reduced immune and inflammatory function, with the majority of research coming from those with sepsis [31–35]. A growing body of evidence suggests mechanically ventilated patients with VAP also exist in this immunosuppressed state.

In a recent prospective observational study, intubated patients with de-compartmentalized VAP displayed a transcriptomic signature indicating relative immune synapse depression, when compared with mechanically ventilated patients who did not develop VAP [36]. Microarray analysis identified immunological signalling pathways expressed differently between patient groups. Gene expression levels were quantified by ddPCR (digital droplet polymerase chain reaction), which is likely to have greater bedside applicability. Depressed genes included those involved in the complement system, cAMP and calcium signalling pathways, which all play a key role in generating the cellular immune response, especially synapsing between antigen presenting cells and lymphocytes. Gene expression levels generally showed an inverse correlation with the clinical pulmonary infection (CPIS) score. These findings suggest patients who develop VAP are immunosuppressed, and ddPCR quantification of genes participating in the immunological synapse may help distinguish patients with VAP from those without.

**Fig. 2:**
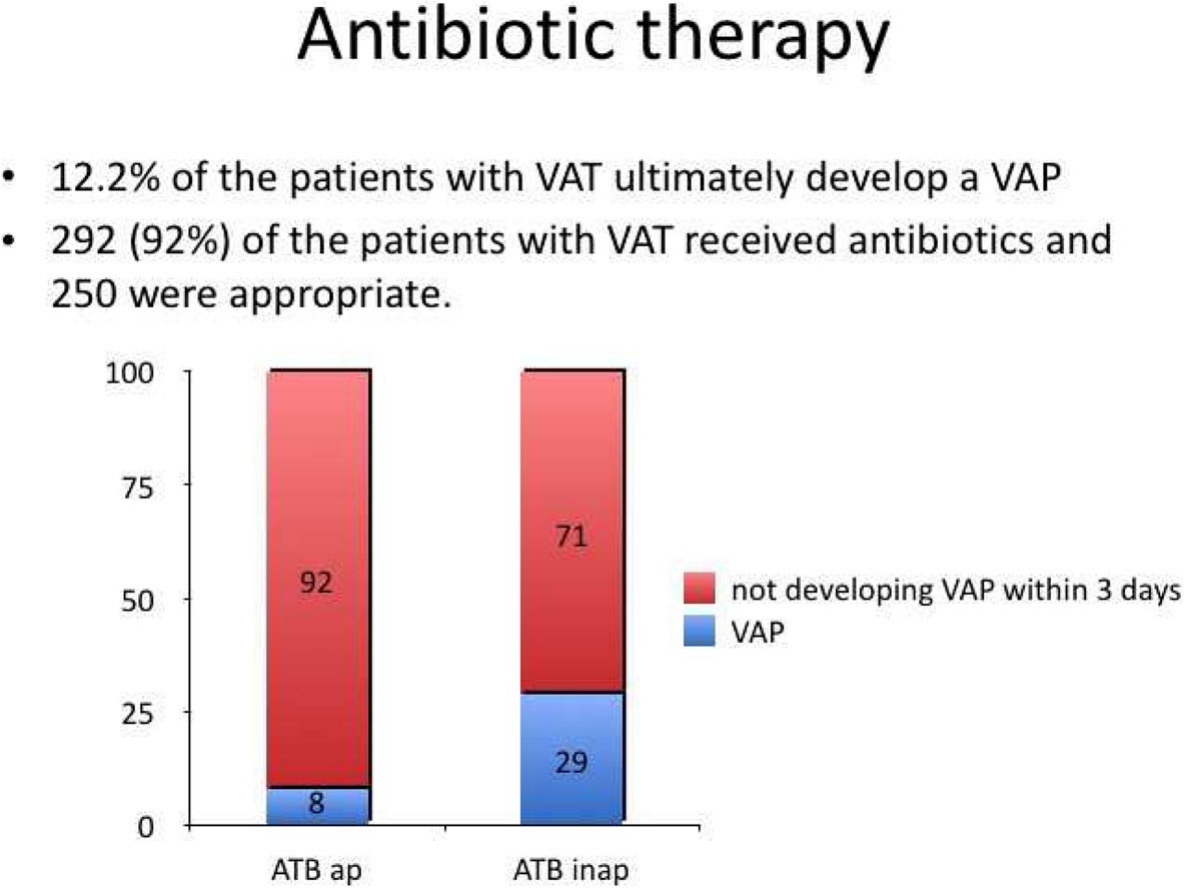
Graph showing appropriate antibiotic therapy for VAT reduced progression to VAP compared with those receiving inappropriate treatment. ATB, antibiotics; VAT, ventilator-associated tracheobronchitis; VAP, ventilator-associated pneumonia. Adapted from the results of the TAVeM study [8]

Discoveries from immune-oriented studies correlate with findings from observational clinical work. VAP has been shown to develop acutely, likely due to an aspiration event, or evolve from pre-existing VAT. The TAVeM observational study found 12.1% of patients with VAT progressed to develop VAP [8]. Patients with VAT who received appropriate antimicrobial therapy had a significantly lower risk of progression to VAP, compared with those receiving inappropriate treatment (19 [8%] of 250 vs. 20 [29%] of 70, *p* < 0.0001) (Fig. 2). These findings are similar to those of a previous prospective study by Nseir and colleagues, who determined 13.9% of patients with VAT subsequently developed VAP, and appropriate antimicrobial therapy was the only factor independently associated with a reduced risk of infective transition [37].

Results from these studies support the hypothesis that patients who progress to VAP from VAT may be in an immunosuppressed state and are reliant on antimicrobial therapy to support dysfunctional cellular immunological pathways. Additionally, previous descriptions promoted to explain divergent disease pathways leading to VAT or VAP might in reality be explained by differences in underlying immune function. Mechanically ventilated patients with an intact immune system may limit pathogenic organisms to colonization or compartmentalized VAT only, while those without may go on to develop de-compartmentalized VAP. This helps explain why VAP exhibits greater inflammation and worse outcomes than those with VAT, suggesting more aggressive antimicrobial therapy is necessary.

This is an exciting area of research, and we may soon use gene expression technology to tailor personalized treatment plans for mechanically ventilated critically ill patients.

### Bedside diagnostics—lab on a chip

Nanotechnology is expected to advance significantly in the coming years, and it is only a matter of time before traditional diagnostic methods are replaced. Techniques are evolving to rapidly and accurately quantify the role of systemic biomarkers, immunological function, and pathogenic organisms on an individual patient basis. Point of care (POC) miniaturized technology is an exciting prospect, which would provide comprehensive analytical data to the ICU clinician in a timely fashion.

**Fig. 3:**
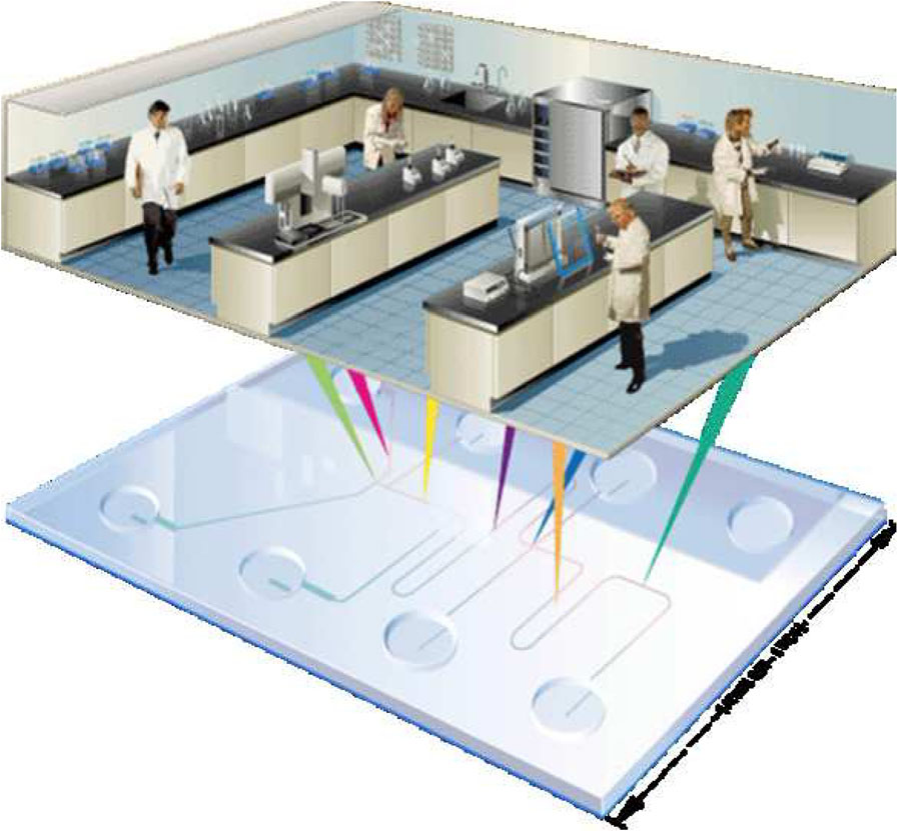
Conceptual image of a point of care lab-on-a-chip device

Microfluidic lab-on-a-chip (LOC) devices are a class of devices that may represent the future of bedside diagnostics for critically ill patients [38–40]. Much of the research in this area relates to patients with cancer, though it is not difficult to imagine the clinical applicability crossing over to the critical care environment. LOC devices are envisioned to integrate and automate multiple laboratory techniques on a miniaturized chip. These devices will be portable, require a small sample volume, and offer rapid detection time in a point-of-care setting (Fig. 3).

A number of ultra-sensitive assays have been developed in the last few years that may facilitate this transfer of diagnostics from the traditional laboratory to the bedside. Such diagnostics are centered on LOC enzyme-linked immunosorbent assays (ELISA) or lab-on-bead nanoparticles.

Timely and accurate POC diagnostics are the future and should enable informed rapid critical care decision-making resulting in improved patient outcomes.

### Personalized medicine

Improved diagnostic pathways and a greater understanding of the role of host-pathogen interaction, systemic biomarkers and immune function should enable progress towards the provision of personalized medicine in the critical care setting.

Progressing to a model of individualized patient assessment and decision-making has the potential to fundamentally alter how we make clinical decisions on a daily basis. Most conventional methods biomarker detection such as microwell plate-based immunoassay and polymerase chain reaction often suffer from high costs, low test speeds and complicated procedures. In addition to clinical assessment, imagine a detailed overview of patient immune function, biomarker trends, and pathogenic organism status, all at the bedside with a high degree of sensitivity and specificity. Mechanically ventilated patients may be stratified according to whether pathogenic organisms are likely to trigger a compartmentalized (VAT) or de-compartmentalized (VAP) immune response. The decision to institute antimicrobial therapy will be more informed, as will the choice of agent and duration of therapy. New disease scoring systems and prognostic calculators are likely to arise as our knowledge and experience with such an approach, ultimately enabling clinicians make more accurate outcome predictions on an individual patient basis.

### Future directions

Our approach to respiratory infections in mechanically ventilated patients will change dramatically in the future. Colonization, VAT and VAP will be seen as a continuum, replacing the current binary model of disease. The research will accurately characterize immune and inflammatory pathways, enhancing our understanding of the host-pathogen interaction, and identify clinically relevant disease-specific biomarkers. Unique immunological “fingerprints” will provide a panel of biomarkers for each individual mechanically ventilated patient, which can be used to anticipate progression to VA-LRTI, whether this will lead to a compartmentalized VAT or a de-compartmentalized VAP, and predict adverse outcomes.

A capacity to predict the onset and severity of VA-LRTI will dramatically alter antimicrobial prescribing in these critically ill patients. Clinicians can logically individualize antimicrobial therapy based on validated biomarkers and unique immune fingerprints, replacing the less than optimal situation currently in place. Choosing single or multi-drug regimens, altering commencement and duration of therapy, and tracking therapeutic response will individualize antimicrobial prescribing. A more logical and objective system for antimicrobial prescribing will curtail the overall usage of antimicrobial agents, and delay or prevent the emergence of resistant pathogens.

Antimicrobial stewardship is clearly of vital long-term importance, but a newer approach to prescribing will also have short-term benefits for individual patients. Early initiation of optimal bespoke antimicrobial therapy, where the intensity and duration of therapy is tailored to clinical, immune and biomarker response, will reduce progression to overwhelming infection and improve survival.

New platforms not only enable better provide attractive characteristics such as label-free detection and improved sensitivity with the implementation of the integration of various novel detection techniques but also sample preparation, chemical manipulation and reaction, high-throughput and portability. All of these aforementioned features can potentially improve the performance for diagnosis and ultimately impact the biomarker’s detection process. Although considered dramatic, a new future of respiratory infections in mechanically ventilated patients will facilitate early intervention and optimization of antimicrobial therapy. This approach will benefit the individual patient, and through improved antimicrobial stewardship, society as a whole.

## Conclusions

Clinician understanding of the host-pathogen interaction during mechanical ventilation is evolving, and it is time to recognize a continuum exists between colonization of the lower respiratory tract, VAT and VAP. This paradigm shift is underpinned by the dissemination hypothesis, which acknowledges the role of the immune and inflammatory systems in determining host response to pathogenic organisms in the lower respiratory tract. Personalized medicine centered on bedside diagnostics assisting rapid clinical decision is on the horizon. The approach to respiratory infections is evolving, and while the future is not yet clear, it is certainly exciting.

No two individual patients are exactly the same, and we look forward to the day our diagnostic and therapeutic strategy can take this into account.
